# Random Number Generation in Adults With Dyslexia: Further Evidence of Dyslexia‐Related Executive Function Difficulties

**DOI:** 10.1002/dys.70030

**Published:** 2026-03-16

**Authors:** Emmanuella Joy Osofisan, Elisa Carrus, James H. Smith‐Spark

**Affiliations:** ^1^ London South Bank University London UK

**Keywords:** adults, developmental dyslexia, executive function, random number generation, strategy selection, supervisory attentional system

## Abstract

Growing evidence indicates that people with dyslexia have executive function deficits. The current study used a random generation task as a novel way to investigate executive function in adults with dyslexia. Participants (total *N* = 54) were asked to produce an unpredictable sequence of 100 digits verbally. Over the course of the task, the dyslexia group's performance improved gradually, while the control group's performance remained stable. An analysis by 25‐digit quartiles showed that the group with dyslexia performed better in the middle to end of the task when compared with their performance earlier in the task. This result suggests that the dyslexia group initially struggled with the executive demands due to task novelty and difficulties with instantiating appropriate strategies. Over time, the group with dyslexia improved and performed better than the group without dyslexia by the end of the task. These initial difficulties are suggestive of dyslexia‐related Supervisory Attentional System (SAS) dysfunction, with the SAS in dyslexia progressively operating more smoothly in its continuous monitoring and control of responses. As well as contributing to theoretical discussions about the role of executive function in dyslexia, the results have implications for supporting people with dyslexia when encountering new tasks.

## Summary

- Random number generation needs executive control to prevent non‐random responses.
- Dyslexic executive function has not been tested using random generation before.
- In an adult sample, dyslexia status interacted with some aspects of randomness performance over time.
- Initial problems with task novelty suggest dyslexia‐related executive difficulties.
- Dyslexic performance improved with more practice, with educational implications.

## 1 Introduction

Developmental dyslexia (henceforth, dyslexia) is a neurodevelopmental disorder that is characterised by an impairment in reading and spelling acquisition; it occurs across a range of intellectual abilities with varying degrees of severity and impact (Rose 2009; Snowling and Hulme 2024; Snowling et al. 2020). The prevalence rate is estimated to be 5% to 10% of the world population (e.g., Olusanya et al. 2023; Siegel 2006). The phonological deficit hypothesis (Vellutino 1979) is one of the most widely held theories of dyslexia. It posits that people with dyslexia have a specific impairment in the representation, storage, and retrieval of phonemes (see reviews by Vellutino et al. 2004, and Castles and Friedmann 2014). From this theoretical perspective, dyslexia‐related difficulties in learning to read are largely explained by problems with learning and processing grapheme‐phoneme correspondences. Nevertheless, the difficulties faced by individuals with dyslexia are not limited to phonological processing. For example, it has been found that children with dyslexia exhibit impaired working memory (e.g., Ackerman and Dykman 1993; Helland and Asbjørnsen 2004; Menghini et al. 2011; Wang et al. 2022). They also show poorer performance on a range of motor skill tasks (Decarli et al. 2024; Fawcett et al. 1996; Thompson et al. 2015), balancing tasks (Getchell et al. 2007; Nicolson and Fawcett 1990), time estimation tasks (Casini et al. 2018; Nicolson et al. 1995), and in carrying out more than one task at once (Augur 1985; Nicolson and Fawcett 1990). While it is acknowledged that there is a core phonological processing deficit which is characteristic of dyslexia, the wider range of dyslexic deficits highlighted above could be indicative of a broader executive functioning problem. Hitherto, executive function in children with dyslexia has been studied extensively (for meta‐analytic reviews, see Booth et al. 2010, and Lonergan et al. 2019), while there are relatively far fewer studies on cognition in adults with dyslexia, especially in regard to executive functioning (Smith‐Spark et al. 2016). Moreover, the study of adults with dyslexia is necessary because, first, dyslexia‐related cognitive deficits persist in adulthood (e.g., Fawcett 2014; McLoughlin et al. 1994) and, second, the cognitive demands faced by an adult are likely to be very different to those placed on a child (see, e.g., Garon et al. 2008). The present study, therefore, sought to explore executive function in adults with dyslexia using a well‐established test of executive function, random number generation, which had not been used previously in dyslexia research.

Executive function represents a collection of top‐down mental processes that are necessary for any activity that requires concentration and attention (Diamond 2020; Lezak 1982; Miyake et al. 2000). In consequence, executive function is effortful and taxes attentional resources, rather than being, automatic and undemanding (Diamond 2013). These executive processes are responsible for higher‐order, goal‐directed capabilities such as problem solving, planning, sequencing actions, processing information selectively, retaining task‐relevant information, and adapting responses according to changes in environmental demands (e.g., Welsh et al. 1991). At its very core, Miyake et al. (2000) conceptualised executive function as having a tripartite structure, comprising the overarching and moderately intercorrelated, yet clearly separable, executive abilities of updating, inhibition, and set shifting. Updating is responsible for monitoring and coding relevant information for the task at hand and then, in the light of new information, refreshing the items stored in working memory (Morris and Jones 1990). Inhibition reflects the ability to deliberately inhibit or delay dominant, prepotent or automatic responses to achieve a goal (Friedman and Miyake 2004; Miyake et al. 2000; Nigg 2000). Set shifting is called upon when needing to move flexibly between multiple tasks, mental sets or operations (Monsell 1996, 2003). The three core executive functions are regarded as the building blocks of the more complex executive functions such as planning, reasoning, and problem solving (Collins and Koechlin 2012; Gamino et al. 2022; Lunt et al. 2012). Fisk and Sharp (2004) added a further core executive function termed verbal fluency or access to long‐term memory. Fluency can be defined as the ability to generate verbal or non‐verbal items according to certain rules within a defined and short period of time (e.g., Phillips et al. 2002; Troyer et al. 1997).

Random generation can be used to assess executive abilities. Indeed, Phillips (1997) has proposed that the random number generation task has high face validity as a test of executive function because the task demands are very novel and the task is cognitively effortful. The most common random generation task is to ask participants to verbally produce a sequence of digits or letters in as random a fashion as possible (e.g., A. Baddeley 1996). The collective involvement of executive function in random number generation has been documented. First, Miyake et al. (2000) examined the correlational structure of performance between random generation and simple executive function tasks; second, Cooper et al. (2012) examined the way in which different secondary executive functioning tasks might interfere with random letter generation performance (compared with baseline performance where there was no secondary task). Inhibition is needed for the suppression of stereotyped responses such as habitual counting (Baddeley et al. 1998; Oomens et al. 2015). Updating is required for the monitoring of recent responses and response distribution (Biesaga and Nowak 2024; Jahanshahi et al. 1998; Miyake et al. 2000). Set shifting has been argued to be involved in the switching of response strategies, such as counting up by three and then saying an even number (Cooper 2016), and there are similarities between verbal fluency and random number generation in terms of the production of appropriate responses according to the rules specified in the task instructions (Baddeley et al. 1998; Fisk and Sharp 2004; Jahanshahi et al. 1998).

As a more general account of executive function, Norman and Shallice's Model of the Control of Action (1986) proposes that there are two complementary processes involved in the control of action. Namely, these are contention scheduling and the SAS, which are recruited depending on the environment and task demands. Specifically, in routine situations, there are existing schemata that channel behaviour into automatic well‐learnt habitual response patterns—this is known as contention scheduling (see also Cooper and Shallice 2000). In novel situations, the SAS is called upon to exert executive top‐down control to guide behaviour. It acts as a monitoring system and it selectively excites or inhibits the schemata within the contention scheduling system. Random number generation has been argued to require the constant intervention of the SAS to override strategies, such as counting, in favour of suitably random strategies, such that the automatically generated non‐random responses are “filtered out” (A. D. Baddeley 1986; Jahanshahi et al. 2006; Towse 1998). According to Baddeley (1996), see also Baddeley et al. (1998), random number generation involves two steps. In step one, a putative response is generated through the retrieval of response schemata (e.g., counting upward by two and then saying a low odd number). In step two, the response is evaluated in light of recent responses, to determine whether or not the identified response is “random”. If the response passes this evaluation, then it is produced. If, however, the response is deemed to be non‐random or not random enough (and if time and cognitive resources allow), then the production of the response is inhibited and there is then a switch to a different response schema, with a new response being generated in an iterative manner.

Random generation performance is not underpinned by one single construct. Indeed, Towse and Valentine (1997) identified four underlying factors: equality of response usage, prepotent associates, and long and short repetitions. The equality of response usage is the degree to which each response (e.g., digit) occurs as equally often as the others. The prepotent associates factor reflects the inability to suppress stereotyped responses, such as counting. The short and long repetitions factors represent, respectively, the tendency to refrain from repetition within a small sequence length or a long sequence length. Because of these underlying factors, Towse and Valentine (1997) identified 16 different measures to score random number generation performance; five of these were used in the current study and are detailed in the Method.

Dyslexia‐related executive function difficulties have been documented in both children (see reviews by Booth et al. 2010, and Lonergan et al. 2019; López‐Zamora et al. 2025) and, less extensively, in adults (e.g., Brosnan et al. 2002; Farah et al. 2021; Smith‐Spark et al. 2016, 2017). A study that is of direct relevance to the current investigation was conducted by Smith‐Spark and Fisk (2007). This investigated spatial working memory in adults with and without dyslexia. Although Smith‐Spark and Fisk found no overall group difference in performance, there was a significant interaction between group membership and task performance in the first half and second half of the task. Specifically, the group with dyslexia had lower recall accuracy than the group without dyslexia in the first half of the task, but their performance improved to a level that was equivalent to the group without dyslexia in the second half. By administering a spatial (and, thus, non‐verbal) working memory task, Smith‐Spark and Fisk demonstrated that the difficulties experienced by the group with dyslexia could not be accounted for by a phonological processing problem. Smith‐Spark and Fisk appealed to the SAS (Norman and Shallice 1986) as an explanation for this pattern of results that they obtained, proposing that the participants with dyslexia were initially slower to instantiate new schemata for efficient production strategies in response to a novel task. Other research (e.g., Bacon et al. 2007; Bacon et al. 2013; Meltzer 1991; Reiter et al. 2005; Torgeson and Goldman 1977) has also provided evidence that is indirectly supportive of a dyslexia‐related impairment to the SAS and broad difficulties with adopting appropriate strategies when a task with novel demands is first encountered (see Smith‐Spark and Gordon 2022).

Random number generation is, thus, a key aspect of executive function, with high levels of novelty relating to its task demands. However, to the best of the authors' knowledge, it has not yet been explored in individuals with dyslexia. The aim of the current study was, therefore, to investigate random number generation performance in adults with dyslexia. In the current study, the participants were asked to produce an unpredictable sequence of 100 digits verbally. Because random number generation is a complex measure of executive function (e.g., Miyake et al. 2000; Peters et al. 2007), several measures were used as an index of randomness of the responses produced—some of these measures tapped a core executive function, while others provided an indication of global randomness. If a dyslexia‐related deficit were to be observed in random generation behaviour, this would provide further evidence in favour of executive function problems in dyslexia and further highlight the need for theoretical accounts of dyslexia to be able to explain the associated deficits in executive function within their frameworks. It was hypothesised that the adults with dyslexia would generate sequences that were less random than those of the adults without dyslexia. Based on the findings of Smith‐Spark and Fisk (2007), it was further hypothesised that the performance of the group with dyslexia would be less random in the earlier stages of the task, subsequently improving by the later stages of the task to a level at least equivalent to that of the group without dyslexia.

## 2 Method

### 2.1 Participants

Participants were recruited, through opportunistic sampling, from the general public and university population at the authors' host institution and the first author's affiliate institution. They were self‐declared native English speakers and received remuneration in the form of either shopping vouchers or course credits. Potential participants with a diagnosis of ADHD were not permitted to take part in the study due to the executive function difficulties associated with the condition (e.g., Barkley 1997; Kaga et al. 2020; Lambek et al. 2011). Participants were divided into two groups, namely individuals with dyslexia and individuals without dyslexia, initially based on individuals' self‐declared dyslexia status (‘I have dyslexia’ vs. ‘I do not have dyslexia’). Documentary evidence in support of the diagnosis for participants with dyslexia was also shown to the researcher at the start of the testing session; these reports from educational psychologists also indicated no co‐occurring neurodevelopmental conditions such as ADHD or dyspraxia. Further to this, dyslexia screening tests for spelling and reading were administered to all the participants, as detailed below.

The Nonsense Word Reading Test is taken from the Dyslexia Adult Screening Test (DAST; Fawcett and Nicolson 1998). The participants were requested to read a passage of text containing real words and nonsense words. Tests of reading that contain nonsense words are argued to be highly sensitive to the presence of dyslexia in that nonsense words must be decoded through grapheme‐phoneme conversion (Gross‐Glenn et al. 1990; Hatcher et al. 2002). Reading speed and accuracy were both recorded to produce a DAST Nonsense Word Reading Test raw score, where lower scores reflected a greater likelihood of dyslexia. Depending on age, all participants in the control group had to score above the age‐normed region of 83–87, which was the cut‐off point for identification as being ‘at risk’ of dyslexia.

The spelling component of the Wechsler Objective Reading Dimensions (WORD; Wechsler 1993) was administered to provide a spelling age for each participant. The researcher read aloud a series of words for the participant to spell, each followed by a sentence in which the target word was contextualised, and then the target word was repeated. As the task progressed, the words became increasingly difficult to spell and, in line with the test instructions, if six consecutive incorrect responses were made, the test was ended by the researcher. A WORD spelling raw score between 42 and the maximum score of 50 corresponded to a spelling age of greater than 17 years, indicating a spelling age in the adult range. A score in this range was required for participants to be included in the control group (see Appendix A for the full range of raw scores for the group with dyslexia and the group without dyslexia). To ensure that the participant groups were well matched for general cognitive ability independent of the effects of dyslexia, four subtests from the Wechsler Adult Intelligence Scale—Fourth UK Edition (WAIS‐IV; Wechsler 2010) were administered. The Block Design, Vocabulary, Comprehension, and Picture Completion subtests are not sensitive to the effects of dyslexia (Turner 1997) and, therefore, provide a fair comparison of general intellectual ability between the two groups. A short‐form IQ for all participants was calculated based on the participant's scaled scores from the four subtests, using Turner's formula.

Based on the above approach, 26 individuals were allocated to the group of adults with dyslexia (five males, 21 females, mean age = 25.46 years, SD = 6.72, range = 18–39 years) and 28 to the group of adults without dyslexia (nine males, 19 females, mean age = 23.43 years, SD = 4.99, range = 18–35 years). The two groups did not differ significantly in age, *t*(52) = −1.27, *p* = 0.21. On the DAST Nonsense Word Reading Test, the group with dyslexia (range = 25–97) scored significantly lower than the group without dyslexia (range = 88–99). On the WORD spelling test, the group without dyslexia attained a significantly higher WORD spelling raw score than the group with dyslexia. Ten of the participants with dyslexia had a spelling age below 17 years, while all the participants in the group without dyslexia had spelling ages in the adult range. The two groups did not differ significantly in short‐form IQ.

Table 1 shows the participant demographics and mean short‐form IQ and screening measure scores.

**TABLE 1:** Mean scores for the screening measures and short‐form IQ (standard deviations in parentheses).

		Group with dyslexia	Group without dyslexia	Independent‐samples *t*‐test			
				*t*	df	*p*	Cohen's *d*
*N*		26 (5 males, 21 females)	28 (9 males, 19 females)	—	—	—	—
Age (years)		25.46 (6.72) 18–35	23.43 (4.99) 18–35	−1.27	52	0.21	—
	Range						
WAIS‐IV short‐form IQ		94.30 (7.05) 82.00–108.00	96.00 (9.17) 79.20–116.50	< 1	52	0.449	—
	Range						
DAST nonsense word reading score		80.81 (18.16) 25–97	96.54 (2.67) 88–99	4.37	26.01*	0.001	1.24
	Range						
WORD spelling test raw score		40.81 (4.95) 27–48	46.00 (2.47) 42–50	4.94	52	0.001	1.34
	Range						

* Levene's test was significant, so equal variances were not assumed.

### 2.2 Materials

The Random Number Generation task (Towse 1998) was employed to assess executive function. A digital metronome producing “clicks” at a rate of one per second was used to encourage participants to generate random digits at a steady pace. The RgCalc computer program (Towse and Neil 1998) was used to quantify the degree of randomness of the participants' sequences.

### 2.3 Design

The data were analysed using two‐way mixed ANCOVAs wherein dyslexia status (adults with dyslexia versus adults without dyslexia) was the between‐participants factor and quartile (1, 2, 3, & 4) was the within‐participants factor. Short‐form IQ was entered as the covariate. Five separate ANCOVAs were carried out for each of the dependent variables. The degree of randomness evident in the generated number sequences was measured according to the following indices of randomness: redundancy, median gap repetition, adjacency, turning point index, runs, and random number generation (RNG). The random number sequences generated by each participant were also divided into quartiles of 25 responses so that the various measures of randomness could be used to assess performance over the duration of the experiment. Each of the randomness measures is now described in detail.

#### 2.3.1 Redundancy

Redundancy (or R) is a linear transformation of Shannon's entropy (Shannon 1948) of the multiset of elements in sequence (Cooper 2016). The redundancy measure indicates response equality; that is, the degree to which each response (i.e., a digit) occurs as equally often as the others. The lower the redundancy score, the greater the randomness of performance. Redundancy scores range from 0 to 100, with 0 indicating that each response is equally frequent and 100 indicating complete redundancy (being the same single response repeated throughout). Redundancy is argued to tap the updating executive function and reflects a behavioural strategy of cycling through the responses as evenly as possible by monitoring previous output (Ginsburg and Karpiuk 1994; Rabinowitz 1970).

#### 2.3.2 Median Gap Repetition

This measure gives an index of the median number of responses generated until any given digit occurs again in the sequence (Proios et al. 2008). Median gap repetition is, thus, determined by counting the number of digits in the sequence that arise between the production of two identical digits, then calculating the median of that figure (Peters et al. 2007). Median gap repetition has been found to load highly on the equality of response usage component of random number generation performance (Towse and Valentine 1997) and is considered to tap the executive function of updating (Miyake et al. 2000). In a random number generation task with 10 response alternatives, the gap repetition would range from zero to 10. A high median gap repetition score is indicative of frequent cycling—reflecting a strategy wherein the respondent attempts to evenly cycle through all digits before generating a repeat digit; this strategy requires the careful monitoring of previous output. While cycling reflects an ability to maintain several digits in working memory and then generate a digit that differs from these numbers, an over‐reliance on cycling signifies an overly simplistic and rigid random generation behaviour that does, in fact, lead to a deviation from randomness because the strategy is itself systematic.

#### 2.3.3 Adjacency

The adjacency (or A) measure of randomness provides an index of the frequency of adjacent response pairs (e.g., “6 and 7” or “3 and 2”). Adjacency scores range from zero to 100, with zero signifying that there were no neighbouring pairs in the sequence and 100 indicating that the entire sequence consisted of neighbouring pairs. The lower the A score, the greater the degree of randomness that is evident in performance. Adjacency taps the executive function of inhibition. Adjacency reflects the tendency to count upwards or downwards in steps of one. This tendency is symptomatic of the inability to suppress stereotyped responses. Adjacency loads highly on Towse and Valentine's (1997) prepotent associates factor.

#### 2.3.4 Turning Point Index

The number of turning points, or changes between ascending and descending sequences, is known as the turning point index (Azouvi et al. 1996; Kendall 1976). For example, the sequence, “1, 3, 8, 9, 6, 2” contains one turning point at “9”, after which the sequence begins to descend. The turning point index value is given as a percentage. A value of 100% signifies a theoretically perfect random distribution of responses (Towse and Neil 1998). Thus, values greater than 100% mean that too many turning points were made in the sequence, whereas values less than 100% indicate that too few turning points have been made. Azouvi et al. (1996) reported that a group of patients with closed head injury attained significantly lower turning point index than a control group. This finding could be interpreted as indicating an over‐reliance on a disadvantageous runs strategy where participants produce unidirectional uninterrupted chains of responses (Azouvi et al. 1996) or a lack of cognitive flexibility to switch between ascending and descending schemata (see Cooper 2016). Turning point index loads highly on the prepotent associates component of random number generation performance and is argued to capture the inhibition executive function (Proios et al. 2008; Towse and Neil 1998).

#### 2.3.5 Runs

The variability of the number of items (length) in an uninterrupted ascending sequence is signified by the Runs score (Ginsburg and Karpiuk 1994). With Runs loading high on proponent associates, this measure is said to tap the executive function of inhibition (Proios et al. 2008; Towse and Neil 1998). According to Proios et al. (2008), the lower the runs score, the greater the degree of randomness in performance.

#### 2.3.6 RNG

While the redundancy score indicates the distribution of response frequencies, it does not tell us about the randomness of the sequence because the association or dependency between one choice and the next is unknown (Towse and Neil 1998). Evans' Random Number Generation (RNG; Evans 1978), however, is an index of randomness which addresses this weakness. It does so by measuring the distribution of all possible response pairs or digrams (e.g., “1” followed by “5” or “7” followed by “3” etc.); that is, how often any response alternative follows any other response alternative (Towse and Neil 1998). As a result, RNG is a global measure of randomness (Geisseler et al. 2016; Proios et al. 2008) and it correlates most strongly with prepotent associates (Towse and Neil 1998). Scores on the RNG measure range from zero to one. Low RNG scores signify high randomness, such that all possible digrams have been used equally as often. High RNG scores indicate low randomness, meaning that the same digrams have been used repeatedly.

The randomness measures are summarised in Table 2.

**TABLE 2:** Measures of randomness scoring.

Randomness measure	Main executive function utilised	Direction of scores denoting optimal human random generation performance
Redundancy	Updating	Low
Median Gap Repetition	Updating	High
Adjacency	Inhibition	Low
Runs	Inhibition	Low
RNG	Global measure of randomness	Low

On account of the multiple tests conducted, the Benjamini‐Hochberg correction was applied in order to reduce the risk of Type I errors occurring and the false discovery rate was set to 10%.

### 2.4 Procedure

The study was approved by the School of Applied Sciences Ethics Committee at the authors' host institution (reference number: SAS1902). At the beginning of the experiment, the participants received a brief verbal introduction outlining the nature of the study and what the activities would involve. Following this, the participants gave informed consent to take part. In the first session, the dyslexia screening tasks and short‐form IQ test were administered; the duration of this session was approximately one hour. The participants were then given a 15‐min rest break. Next, in a counterbalanced order, the random number generation task was administered alongside three other tasks for a different study, which are not documented here. This second session lasted approximately 25 min.

In the Random Number Generation task (Towse 1998), the participants were required to generate a sequence of 100 random numbers. At the start of the task, the participants were told to imagine a hat containing 10 balls with a single number from 1 to 10 printed on each ball. In the hypothetical scenario, the participant dipped his or her hand into a hat, pulled out a ball, read the number, replaced the ball in the hat, shuffled the hat, and then repeated the process. Following this, the participants were required to verbally generate a random digit from one to 10 at the onset of an auditory click. The click was presented 100 times, with one click being played every second.

At the end of testing, all participants received a verbal debrief, followed by a written debrief.

## 3 Results

To assess random number generation performance, five two‐way mixed ANCOVAs were conducted on each randomness measure.

Assumptions for the ANCOVAs were first checked. An additional measure of randomness, Turning Point Index, was excluded from the analyses because the assumption of homogeneity of regression slopes was violated as there was a significant interaction term between dyslexia status and short‐form IQ.

To meet the assumption of normality, a square root transformation of Redundancy in Quartiles 1–4, and Runs in Quartiles 1 and 2 was applied so that the data for each combination of the between‐participants and within‐participants factors were approximately normally distributed.

For each combination of the between‐participants and within‐participants factors, there was a linear relationship between the dependent variable and the covariate as indicated by the matrix scatterplots.

The assumption of homogeneity of variance was met across all groups except for Redundancy in Quartile 2 and Median Gap Repetition in Quartile 2.

Sphericity was assumed in all models except Runs, so the Greenhouse–Geisser correction was used for the latter dependent variable.

The key finding was that the adults with dyslexia performed significantly worse than the adults without dyslexia on Runs in the first quartile and that the adults with dyslexia performed significantly better than the adults without dyslexia on RNG performance in the third and fourth quartiles. The results of the remaining ANCOVAs all showed non‐significant main effects and non‐significant interactions, as detailed below. The descriptive statistics are displayed in Tables 3, 4, 5, 6, 7.

**TABLE 3:** Descriptive statistics for Redundancy across quartiles.

Redundancy	Mean score (SD) of the group with dyslexia	Mean score (SD) of the group without dyslexia	Range of the group with dyslexia	Range of the group without dyslexia	Skewness (SE) of the group with dyslexia	Skewness (SE) of the group without dyslexia	Kurtosis (SE) of the group with dyslexia	Kurtosis (SE) of the group without dyslexia
Quartile 1	4.29 (2.45)	3.86 (2.70)	0.87‐ 9.13	0.87‐ 9.13	1.09 (0.50)	1.51 (0.51)	0.13 (0.97)	1.62 (0.99)
Quartile 2	5.81 (5.20)	4.00 (2.63)	0.87‐ 16.59	0.87‐ 6.60	1.50 (0.50)	1.39 (0.51)	1.23 (0.97)	1.37 (0.99)
Quartile 3	4.49 (3.31)	4.60 (2.99)	0.87‐ 13.16	1.46‐ 8.81	2.00 (0.50)	1.00 (0.51)	5.38 (0.97)	0.15 (0.99)
Quartile 4	4.31 (3.15)	4.15 (2.10)	0.87‐ 10.51	0.87‐ 8.81	1.64 (0.50)	0.82 (0.51)	3.75 (0.97)	0.68 (0.99)

**TABLE 4:** Descriptive statistics for Median Gap Repetition across quartiles.

Median Gap Repetition	Mean score (SD) of the group with dyslexia	Mean score (SD) of the group without dyslexia	Range of the group with dyslexia	Range of the group without dyslexia	Skewness (SE) of the group with dyslexia	Skewness (SE) of the group without dyslexia	Kurtosis (SE) of the group with dyslexia	Kurtosis (SE) of the group without dyslexia
Quartile 1	7.29 (1.02)	7.43 (1.18)	6.00–9.00	6.00–9.00	−0.13 (0.50)	−0.25 (0.51)	−0.67 (0.97)	−1.14 (0.99)
Quartile 2	7.56 (1.43)	7.32 (0.94)	6.00–10.00	5.00–9.00	−0.36 (0.50)	−0.36 (0.51)	−0.60 (0.97)	1.07 (0.99)
Quartile 3	6.94 (1.43)	7.59 (1.28)	6.00–10.00	5.50–10.00	1.04 (0.50)	0.07 (0.51)	0.75 (0.97)	−1.05 (0.99)
Quartile 4	7.25 (1.41)	7.11 (1.03)	4.50–10.00	5.00–9.00	−0.35 (0.50)	−0.07 (0.51)	0.42 (0.97)	−0.61 (0.99)

**TABLE 5:** Descriptive statistics for Adjacency across quartiles.

Adjacency	Mean score (SD) of the group with dyslexia	Mean score (SD) of the group without dyslexia	Range of the group with dyslexia	Range of the group without dyslexia	Skewness (SE) of the group with dyslexia	Skewness (SE) of the group without dyslexia	Kurtosis (SE) of the group with dyslexia	Kurtosis (SE) of the group without dyslexia
Quartile 1	17.23 (11.01)	12.71 (9.62)	0.00–32.00	0.00–36.00	0.03 (0.50)	0.53 (0.51)	−0.66 (0.97)	−0.77 (0.99)
Quartile 2	18.92 (12.83)	14.14 (10.30)	0.00–52.00	0.00–32.00	0.95 (0.50)	0.33 (0.51)	1.26 (0.97)	−1.21 (0.99)
Quartile 3	17.85 (12.56)	13.00 (11.55)	0.00–36.00	0.00–36.00	0.12 (0.50)	0.87 (0.51)	−0.84 (0.97)	−0.42 (0.99)
Quartile 4	15.23 (10.97)	14.14 (8.67)	0.00–32.00	4.00–32.00	0.12 (0.50)	0.67 (0.51)	−1.37 (0.97)	−0.45 (0.99)

**TABLE 6:** Descriptive statistics for Runs across quartiles.

Runs	Mean score (SD) of the group with dyslexia	Mean score (SD) of the group without dyslexia	Range of the group with dyslexia	Range of the group without dyslexia	Skewness (SE) of the group with dyslexia	Skewness (SE) of the group without dyslexia	Kurtosis (SE) of the group with dyslexia	Kurtosis (SE) of the group without dyslexia
Quartile 1	0.98 (0.47)	0.65 (0.38)	0.27‐ 2.07	0.00‐ 1.92	0.83 (0.50)	1.34 (0.51)	0.27 (0.97)	3.02 (0.99)
Quartile 2	1.01 (0.59)	0.72 (0.44)	0.13‐ 2.83	0.12‐ 2.25	1.36 (0.50)	1.60 (0.51)	3.29 (0.97)	4.06 (0.99)
Quartile 3	0.68 (0.35)	0.90 (0.61)	0.12‐ 1.27	0.00‐ 3.13	−0.19 (0.50)	1.56 (0.51)	−0.36 (0.97)	4.12 (0.99)
Quartile 4	0.82 (0.51)	0.77 (0.43)	0.00‐ 1.82	0.13‐ 1.77	0.35 (0.50)	0.54 (0.51)	−0.69 (0.97)	−0.76 (0.99)

**TABLE 7:** Descriptive statistics for RNG across quartiles.

RNG	Mean score (SD) of the group with dyslexia	Mean score (SD) of the group without dyslexia	Range of the group with dyslexia	Range of the group without dyslexia	Skewness (SE) of the group with dyslexia	Skewness (SE) of the group without dyslexia	Kurtosis (SE) of the group with dyslexia	Kurtosis (SE) of the group without dyslexia
Quartile 1	0.18 (0.09)	0.16 (0.08)	0.00‐ 0.37	0.00‐ 0.35	0.07 (0.50)	0.24 (0.51)	−0.37 (0.97)	0.14 (0.99)
Quartile 2	0.20 (0.10)	0.16 (0.09)	0.00‐ 0.38	0.00‐ 0.34	0.18 (0.50)	0.67 (0.51)	−0.30 (0.97)	0.73 (0.99)
Quartile 3	0.13 (0.06)	0.17 (0.08)	0.00‐ 0.26	0.06‐ 0.34	0.32 (0.50)	0.09 (0.51)	−0.21 (0.97)	−0.14 (0.99)
Quartile 4	0.14 (0.08)	0.19 (0.10)	0.00‐ 0.31	0.00‐ 0.41	0.15 (0.50)	0.67 (0.51)	0.01 (0.97)	0.75 (0.99)

The mixed ANCOVA for Redundancy showed that the effect of dyslexia status was not significant, *F*(1, 51) = 1.40, *p* = 0.51, *η*_*p*_^*2*^ = 0.01. The effect of quartile was not statistically significant, *F*(3, 153) = 0.44, *p* = 0.24, *η*_*p*_^*2*^ = 0.03. The covariate of short‐form IQ was also not significant, *F*(1, 51) = 0.20, *p* = 0.65, *η*_*p*_^*2*^ = 0.004. Dyslexia status and quartile were not found to interact significantly, *F*(3, 153) = 1.15, *p* = 0.33, *η*_*p*_^*2*^ = 0.02.

There was no effect of dyslexia status on Median Gap Repetition, *F*(1, 51) = 0.23, *p* = 0.64, *η*_*p*_^*2*^ = 0.004. Quartile also did not have a significant effect, *F*(3, 153) = 1.44, *p* = 0.23, *η*_*p*_^*2*^ = 0.03. The covariate of short‐form IQ was also not significant, *F*(1, 51) = 1.16, *p* = 0.69, *η*_*p*_^*2*^ = 0.003. There was no significant dyslexia status × quartile interaction, *F*(3, 153) = 1.78, *p* = 0.15, *η*_*p*_^*2*^ = 0.03.

The effect of dyslexia status on Adjacency was non‐significant, *F*(1, 51) = 2.33, *p* = 0.13, *η*_*p*_^*2*^ = 0.04. The effect of quartile was also non‐significant, *F*(3, 153) = 1.99, *p* = 0.12, *η*_*p*_^*2*^ = 0.04. The short‐form IQ covariate was not significant, *F*(1, 51) = 0.26, *p* = 0.61, *η*_*p*_^*2*^ = 0.005. Dyslexia status and quartile did not interact significantly, *F*(3, 153) = 1.15, *p* = 0.12, *η*_*p*_^*2*^ = 0.04.

**FIGURE 1:**
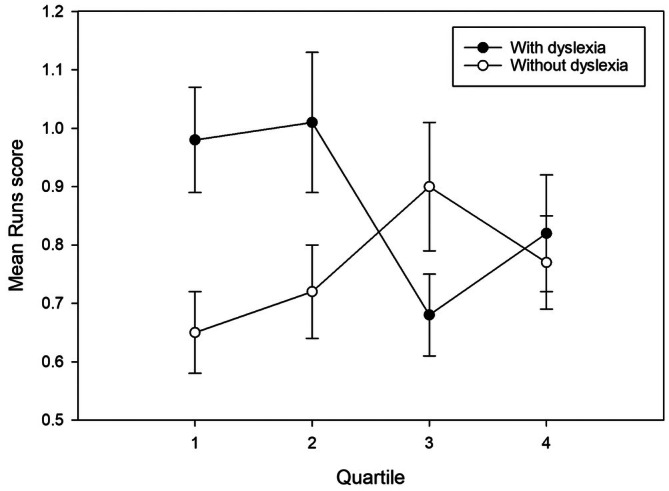
Runs scores across quartiles in adults with dyslexia and adults without dyslexia. Lower scores indicate greater randomness.

For Runs, the effect of dyslexia status was not significant, *F*(1, 51) = 0.76, *p* = 0.39, *η*_*p*_^*2*^ = 0.02. The effect of quartile was not significant, *F*(2.47, 125.91) = 0.86, *p* = 0.45, *η*_*p*_^*2*^ = 0.02. The covariate effect of short‐form IQ was not significant, *F*(1, 51) = 1.20, *p* = 0.28, *η*_*p*_^*2*^ = 0.02. However, in this case, the dyslexia status × quartile interaction was found to be statistically significant, *F*(2.47, 125.91) = 3.07, *p* = 0.04, *η*_*p*_^*2*^ = 0.06 (see Figure 1). The Bonferroni post hoc tests revealed that the adults with dyslexia performed significantly worse than the adults without dyslexia in Quartile 1 of Runs, *p* = 0.002, 95% CI [0.08, 0.35]. There was no other significant difference between the adults with dyslexia and the adults without dyslexia in either Quartile 2, *p* = 0.06, 95% CI [−0.01, 0.30], Quartile 3, *p* = 0.15, 95% CI [−0.47, 0.07], or Quartile 4, *p* = 0.72, 95% CI [−0.21, 0.31].

**FIGURE 2:**
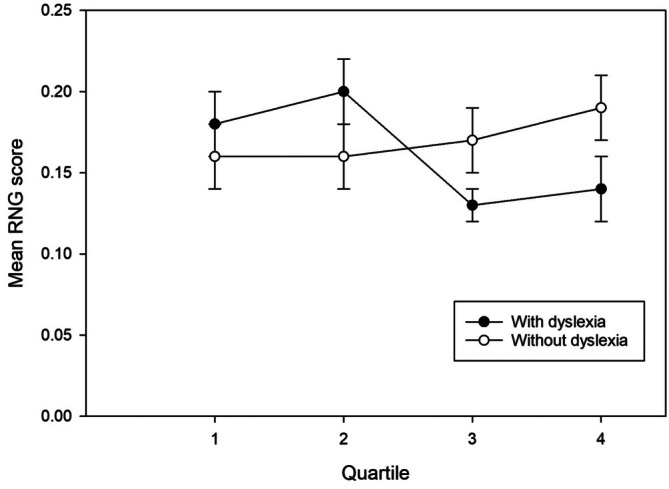
The RNG scores across quartiles in adults with dyslexia and adults without dyslexia. Evans' RNG is a global measure of randomness which is also related to inhibition. Lower scores indicate greater randomness.

The ANCOVA conducted on RNG showed that the effect of dyslexia status was not significant, *F*(1, 51) = 0.61, *p* = 0.44, *η*_*p*_^*2*^ = 0.01. The effect of quartile was also not statistically significant, *F*(3, 153) = 1.31, *p* = 0.27, *η*_*p*_^*2*^ = 0.03. The covariate effect of short‐form IQ was not significant, *F*(1, 51) = 3.21, *p* = 0.08, *η*_*p*_^*2*^ = 0.06. The dyslexia status × quartile interaction was significant, *F*(3, 153) = 4.21, *p* = 0.007, *η*_*p*_^*2*^ = 0.08 (see Figure 2). The Bonferroni post hoc tests showed that there was no significant difference between the adults with dyslexia and the adults without dyslexia in either Quartile 1, *p* = 0.44, 95% CI [−0.03, 0.07], or Quartile 2, *p* = 0.12, 95% CI [−0.01, 0.09]. However, the adults with dyslexia performed significantly better than the adults without dyslexia in Quartile 3, *p* = 0.04, 95% CI [−0.08, −0.003], and Quartile 4, *p* = 0.03, 95% CI [−0.11, −0.01].

## 4 Discussion

The current study used a random number generation task as a novel way to investigate executive function in adults with dyslexia. Random number generation is a complex task that is argued to involve the core executive functions collectively (e.g., A. Baddeley 1996; Jahanshahi et al. 1998; Miyake et al. 2000). The group with dyslexia may have initially struggled with the executive demands of the random number generation task, such that, over the course of the task, the number sequences produced by the group with dyslexia gradually became more random, while the control group's randomness performance remained stable. While group differences were not found across the board, the performance of adults with dyslexia and without dyslexia provided further evidence of dyslexia‐related executive control problems relating to certain aspects of random number generation, particularly in relation to inhibition.

Runs performance is a measure which is argued to tap inhibition (Proios et al. 2008). A key finding of the present study was that the adults with dyslexia performed significantly worse than the adults without dyslexia on Runs in the first quartile. Focusing on the number of runs produced by participants is informative about inhibition and, specifically, reflects the use of a seriation strategy. Seriation is the over‐reliance on the strategy of producing uninterrupted ascending sequences (Ginsburg and Karpiuk 1994). It stems from an inability to suppress stereotyped response schemas (Williams et al. 2002) and this bias is brought about by the overlearned tendency to arrange numbers in their natural order (e.g., “1, 2, 3”; Peters et al. 2007). The higher the Runs score, the less random the sequence that has been produced is. The presence of dyslexia did not have a significant effect on overall Runs performance. However, at a more granular level, when differences in short‐form IQ were statistically controlled, the adults with dyslexia were found to perform significantly worse than the adults without dyslexia in Quartile 1 of Runs; that is, at the very beginning of the task. The examination of Runs scores over the course of the task showed that, as the task progressed, the group with dyslexia became better in generating greater randomness in their responses, while the performance of the group without dyslexia remained relatively stable. Towards the end of the task, the Runs scores produced by the group with dyslexia dropped sharply (i.e., it became more random) in Quartile 3 and further in Quartile 4, where the two groups performed at equivalent levels.

Similarly, this pattern of improved randomness of performance over time was further supported by the analysis by quartiles of RNG scores. Evans' RNG is a global measure of randomness (Geisseler et al. 2016; Proios et al. 2008) with lower RNG scores signifying greater randomness. Performance on RNG scores improved over time, with the largest group difference being observed towards the end of the task. Specifically, when differences in short‐form IQ were statistically controlled, the presence of dyslexia had a significant effect on RNG in Quartiles 3 and 4, such that the adults with dyslexia attained RNG scores that were, on average, lower (i.e., more random) than the controls. There was, however, no effect of dyslexia status on overall RNG performance. As with Runs scores, RNG performance in the group with dyslexia improved as the task progressed and, indeed, the adults with dyslexia outperformed the adults without dyslexia by the end. This pattern of results may be attributable to response to task novelty, an integral characteristic of executive function (e.g., Phillips 1997; Rabbitt 1997), which is greatest when first encountering a task that requires executive function.

Taken together, these results give further weight to the argument for initial executive control difficulties in dyslexia and, more specifically, point towards SAS dysfunction. Consistent with the argument of Smith‐Spark and Fisk (2007), see also Smith‐Spark and Gordon (2022), and Varvara et al. (2014), there would appear to be a dyslexia‐related deficit in the SAS, which can manifest in initial difficulties such as strategy instantiation when confronted with a novel task. However, for individuals with dyslexia, over time, the SAS progressively operates more smoothly in its continuous monitoring of responses and control of the excitation and inhibition of schemata (see Cooper 2016, for more on the operation of the SAS).

In line with the findings of the current investigation, previous research has also provided further evidence of a dyslexia‐related impairment to the SAS and broad difficulties with adopting appropriate strategies when a novel task is first encountered. For example, Smith‐Spark and Fisk (2007) observed how adults with dyslexia had poorer recall accuracy than adults without dyslexia in the first half of a spatial working memory task, but in the second half their performance improved to a level that was equivalent to the group without dyslexia. They explicitly identified a deficit in SAS function as being at the root of the difficulty in coping with novel task demands early on in performance and the relative delay in setting up a schema to deal with them (see also Smith‐Spark and Gordon 2022). Problems with strategy selection have also been observed when adults with dyslexia carry out syllogistic reasoning tasks (Bacon et al. 2007) and spatial working memory span tasks (Bacon et al. 2013). It would likewise appear that dyslexia‐related difficulties exist when executive function is tested using random number generation. When required to generate numbers randomly, the adults with dyslexia were, at first, slower than the adults without dyslexia to instantiate new schemata for efficient production strategies when faced with a very novel task. Successful strategies include the careful monitoring of previous output (for predictability) and frequently switching between a varied set of response schemata, such as counting down by two and then saying a high odd number (A. Baddeley 1996; Baddeley et al. 1998; Williams et al. 2002).

While providing potentially valuable theoretical insights into dyslexia, the current study has some limitations. First, and most generally, the randomness of the sequences generated by participants is evaluated by quantifying the departure from randomness. However, the human concept of random number generation can be subjective and erroneous (Spatt and Goldenberg 1993). For example, Evans (1978) noted that participants tend to avoid immediate repetitions, despite the fact that repetitions can occur by chance. Second, only one form of random generation task was used. Random number generation involves verbal processes (i.e., retrieving, maintaining, and verbally producing digits), so it is not clear the extent to which group differences in performance can be attributed fully to executive control problems rather than phonological processing problems (or a combination of the two; see Smith‐Spark et al. 2017, for a similar argument relating to executive fluency and see Phillips 1997, for the contribution of intelligence and lower‐level cognitive processes to executive function more generally). There would, therefore, be a strong case for future research using additional non‐verbal (or minimally verbal) random generation tasks to address this task impurity issue. More generally, it could be argued that the application of the Model of the Control of Action (Norman and Shallice 1986) to explain performance on laboratory‐based measures is difficult because the stringent and artificial conditions of the laboratory do not reflect real‐world environments (Smith‐Spark and Gordon 2022). Finally, adults with dyslexia may have learned strategies to cope with real‐world difficulties (e.g., Brosnan et al. 2002) and, thus, there is a need for more studies along the lines of the current study, involving tasks with novel demands, more finely grained measures of performance, and high ecological validity.

Based on the results of the current study in which random number generation was investigated, adults with dyslexia would seem to struggle with some aspects of task novelty, especially where inhibition is involved (as indicated by Runs scores). In a similar vein, studies conducted by Gabay et al. (2020), Smith‐Spark et al. (2016), and Wilcockson et al. (2019) also noted dyslexia‐related difficulties with inhibitory control. They all observed that adults with dyslexia performed significantly less accurately than adults without dyslexia when inhibiting prepotent responses. The findings of the current study are also consistent with the argument that there is SAS dysfunction in dyslexia (Smith‐Spark and Fisk 2007; Smith‐Spark and Gordon 2022; Varvara et al. 2014). This dysfunction would seem to lead to difficulties with appropriate strategy instantiation when facing novel task demands (see Phillips 1997), as indicated by the results of the quartiles analyses which indicated lower performance at the start of the random number generation task. The key theoretical implication arising from the current study, therefore, is that executive function difficulties need to be fully incorporated into current explanations of dyslexia. Furthermore, this study has significant implications for learning support and education, since the findings indicate that if given more time for task familiarisation and practice, performance amongst adults with dyslexia greatly recovers to a level commensurate with that of their non‐dyslexic peers. With regard to other accommodations in educational and work settings, the findings of this study may also lend support to the use of greater scaffolding and structuring at the start of a new task. They can also be applied to executive coaching in which a coach, in partnership with the learner, helps to raise an individual's self‐awareness of what must be done to improve their task performance (Doyle and McDowall 2015), thereby providing metacognitive support for dyslexia‐related weaknesses in strategy generation.

## Funding

This study received no external funding.

## Conflicts of Interest

The authors declare no conflicts of interest.

## Data Availability

The raw data that support the findings of this study are available on the Open Science Framework at https://osf.io/rb4qx/?view_only=c4c76e71d0b049cdbec90c10d9f529da.

## Appendix A The Spelling Component of the WORD Raw Scores

**Table:**

Group with dyslexia	Group without dyslexia
44	45
43	49
43	48
38	46
42	47
40	43
41	48
44	47
43	49
42	49
43	48
46	43
45	50
40	42
28	42
27	49
42	45
42	45
43	46
38	43
48	45
40	46
40	46
43	42
44	46
32	49
	47
	43

## References

[dys70030-bib-0001] Ackerman, P. T. , and R. A. Dykman . 1993. “Phonological Processes, Confrontational Naming, and Immediate Memory in Dyslexia.” Journal of Learning Disabilities 26, no. 9: 597–609. 10.1177/002221949302600910. 8283130

[dys70030-bib-0002] Augur, J. 1985. “Guidelines for Teachers, Parents and Learners.” In Children's Written Language Difficulties, edited by M. Snowling , 147–170. NFER‐Nelson.

[dys70030-bib-0092] Azouvi, P. , C. Jokic , M. Van der Linden , N. Marlier , and B. Bussel . 1996. “Working Memory and Supervisory Control After Severe Closed‐Head Injury. A Study of Dual Task Performance and Random Generation.” Journal of Clinical and Experimental Neuropsychology 18, no. 3: 317–337. 10.1080/01688639608408990. 8877617

[dys70030-bib-0003] Bacon, A. M. , S. J. Handley , and E. L. McDonald . 2007. “Reasoning and Dyslexia: A Spatial Strategy May Impede Reasoning With Visually Rich Information.” British Journal of Psychology 98, no. 1: 79–92. 10.1348/000712606X103987. 17227613

[dys70030-bib-0004] Bacon, A. M. , F. B. R. Parmentier , and P. Barr . 2013. “Visuospatial Memory in Dyslexia: Evidence for Strategic Deficits.” Memory 21, no. 2: 189–209. 10.1080/09658211.2012.718789. 22928929

[dys70030-bib-0005] Baddeley, A. 1996. “Exploring the Central Executive.” Quarterly Journal of Experimental Psychology A 49, no. 1: 5–28. 10.1080/027249896392784.

[dys70030-bib-0007] Baddeley, A. D. 1986. Working Memory. Oxford University Press.

[dys70030-bib-0006] Baddeley, A. , H. Emslie , J. Kolodny , and J. Duncan . 1998. “Random Generation and the Executive Control of Working Memory.” Quarterly Journal of Experimental Psychology. A, Human Experimental Psychology 51A, no. 4: 819–852. 10.1080/713755788. 9854442

[dys70030-bib-0008] Barkley, R. A. 1997. “Behavioral Inhibition, Sustained Attention, and Executive Functions: Constructing a Unifying Theory of ADHD.” Psychological Bulletin 121, no. 1: 65–94. 10.1037/0033-2909.121.1.65. 9000892

[dys70030-bib-0009] Biesaga, M. , and A. Nowak . 2024. “The Role of the Working Memory Storage Component in a Random‐Like Series Generation.” PLoS One 19, no. 1: e0296731. 10.1371/journal.pone.0296731. 38241285 PMC10798477

[dys70030-bib-0010] Booth, J. N. , J. M. Boyle , and S. W. Kelly . 2010. “Do Tasks Make a Difference? Accounting for Heterogeneity of Performance of Children With Reading Difficulties on Tasks of Executive Function: Findings From a Meta‐Analysis.” British Journal of Developmental Psychology 28, no. 1: 133–176. 10.1348/026151009x485432. 20306629

[dys70030-bib-0011] Brosnan, M. , J. Demetre , S. Hamill , K. Robson , H. Shepherd , and G. Cody . 2002. “Executive Functioning in Adults and Children With Developmental Dyslexia.” Neuropsychologia 40: 2144–2155. 10.1016/s0028-3932(02)00046-5. 12208010

[dys70030-bib-0012] Casini, L. , C. Pech‐Georgel , and J. C. Ziegler . 2018. “It's About Time: Revisiting Temporal Processing Deficits in Dyslexia.” Developmental Science 21, no. 2: e12530. 10.1111/desc.12530. 28239921

[dys70030-bib-0013] Castles, A. , and N. Friedmann . 2014. “Developmental Dyslexia and the Phonological Deficit Hypothesis.” Mind & Language 29, no. 3: 270–285. 10.1111/mila.12050.

[dys70030-bib-0014] Collins, A. , and E. Koechlin . 2012. “Reasoning, Learning, and Creativity: Frontal Lobe Function and Human Decision‐Making.” PLoS Biology 10, no. 3: e1001293. 10.1371/journal.pbio.1001293. 22479152 PMC3313946

[dys70030-bib-0016] Cooper, R. P. 2016. “Executive Functions and the Generation of “Random” Sequential Responses: A Computational Account.” Journal of Mathematical Psychology 73, no. 1: 153–168. 10.1016/j.jmp.2016.06.002.

[dys70030-bib-0017] Cooper, R. P. , W. Karolina , and E. J. Davelaar . 2012. “Differential Contributions of Set‐Shifting and Monitoring to Dual‐Task Interference.” Quarterly Journal of Experimental Psychology 65, no. 3: 587–612. 10.1080/17470218.2011.629053. 22182315

[dys70030-bib-0015] Cooper, R. , and T. Shallice . 2000. “Contention Scheduling and the Control of Routine Activities.” Cognitive Neuropsychology 17: 297–338. 10.1080/026432900380427. 20945185

[dys70030-bib-0018] Decarli, G. , L. Franchin , and F. Vitali . 2024. “Motor Skills and Capacities in Developmental Dyslexia: A Systematic Review and Meta‐Analysis.” Acta Psychologica 246: 104269. 10.1016/j.actpsy.2024.104269. 38642452

[dys70030-bib-0019] Diamond, A. 2013. “Executive Functions.” Annual Review of Psychology 64: 135–168. 10.1146/annurev-psych-113011-143750. PMC4084861 23020641

[dys70030-bib-0020] Diamond, A. 2020. “Executive Functions.” Handbook of Clinical Neurology 173: 225–240. 10.1016/B978-0-444-64150-2.00020-4. 32958176

[dys70030-bib-0021] Doyle, N. , and A. McDowall . 2015. “Is Coaching an Effective Adjustment for Dyslexic Adults?” Coaching (An International Journal of Theory, Research and Practice) 8, no. 2: 154–168. 10.1080/17521882.2015.1065894.

[dys70030-bib-0022] Evans, F. J. 1978. “Monitoring Attention Deployment by Random Number Generation: An Index to Measure Subjective Randomness.” Bulletin of the Psychonomic Society 12, no. 1: 35–38. 10.3758/BF03329617.

[dys70030-bib-0023] Farah, R. , S. Ionta , and T. Horowitz‐Kraus . 2021. “Neuro‐Behavioral Correlates of Executive Dysfunctions in Dyslexia Over Development From Childhood to Adulthood.” Frontiers in Psychology 12: 708863. 10.3389/fpsyg.2021.708863. 34497563 PMC8419422

[dys70030-bib-0024] Fawcett, A. 2014. “Dyslexia in Adolescent Dyslexics and Students.” In Dyslexia Association of Singapore DAS Handbook 2014, edited by A. Fawcett , 235–240. Dyslexia Association of Singapore.

[dys70030-bib-0025] Fawcett, A. J. , and R. I. Nicolson . 1998. The Dyslexia Adult Screening Test (DAST). Psychological Corporation.

[dys70030-bib-0026] Fawcett, A. J. , R. I. Nicolson , and P. Dean . 1996. “Impaired Performance of Children With Dyslexia on a Range of Cerebellar Tasks.” Annals of Dyslexia 46, no. 1: 259–283. 10.1007/BF02648179. 24234275

[dys70030-bib-0027] Fisk, J. E. , and C. A. Sharp . 2004. “Age‐Related Impairment in Executive Functioning: Updating, Inhibition, Shifting, and Access.” Journal of Clinical and Experimental Neuropsychology 26: 874–890. 10.1080/13803390490510680. 15742539

[dys70030-bib-0028] Friedman, N. P. , and A. Miyake . 2004. “The Relations Among Inhibition and Interference Control Functions: A Latent‐Variable Analysis.” Journal of Experimental Psychology: General 133, no. 1: 101–135. 10.1037/0096-3445.133.1.101. 14979754

[dys70030-bib-0029] Gabay, S. , R. Schiff , and A. Henik . 2020. “Visual and Auditory Interference Control of Attention in Developmental Dyslexia.” Journal of the International Neuropsychological Society 26: 407–417. 10.1017/S135561771900122X. 32238215

[dys70030-bib-0030] Gamino, J. F. , C. Frost , R. Riddle , J. Koslovsky , and S. B. Chapman . 2022. “Higher‐Order Executive Function in Middle School: Training Teachers to Enhance Cognition in Young Adolescents.” Frontiers in Psychology 13: 867264. 10.3389/fpsyg.2022.867264. 35592149 PMC9111740

[dys70030-bib-0031] Garon, N. , S. E. Bryson , and I. M. Smith . 2008. “Executive Function in Preschoolers: A Review Using an Integrative Framework.” Psychological Bulletin 134: 31–60. 10.1037/0033-2909.134.1.31. 18193994

[dys70030-bib-0032] Geisseler, O. , T. Pflugshaupt , A. Buchmann , et al. 2016. “Random Number Generation Deficits in Patients With Multiple Sclerosis: Characteristics and Neural Correlates.” Cortex 82: 237–243. 10.1016/j.cortex.2016.05.007. 27403852

[dys70030-bib-0033] Getchell, N. , P. Pabreja , K. Neeld , and V. Carrio . 2007. “Comparing Children With and Without Dyslexia on the Movement Assessment Battery for Children and the Test of Gross Motor Development.” Perceptual and Motor Skills 105, no. 1: 207–214. 10.2466/pms.105.1.207-214. 17918566

[dys70030-bib-0034] Ginsburg, N. , and P. Karpiuk . 1994. “Random Generation—Analysis of the Responses.” Perceptual and Motor Skills 79: 1059–1067. 10.2466/pms.1994.79.3.1059.

[dys70030-bib-0035] Gross‐Glenn, K. , B. Jallad , L. Novoa , V. Helgren‐Lempesis , and H. A. Lubs . 1990. “Nonsense Passage Reading as a Diagnostic Aid in the Study of Adult Familial Dyslexia.” Reading and Writing: An Interdisciplinary Journal 2, no. 2: 161–173. 10.1007/BF00401800.

[dys70030-bib-0036] Hatcher, J. , M. J. Snowling , and Y. M. Griffiths . 2002. “Cognitive Assessment of Dyslexic Students in Higher Education.” British Journal of Educational Psychology 72: 119–133. 10.1348/000709902158801. 11916468

[dys70030-bib-0037] Helland, T. , and A. Asbjørnsen . 2004. “Digit Span in Dyslexia: Variations According to Language Comprehension and Mathematics Skills.” Journal of Clinical and Experimental Neuropsychology 26, no. 1: 31–42. 10.1076/jcen.26.1.31.23935. 14972692

[dys70030-bib-0038] Jahanshahi, M. , P. Profice , R. G. Brown , M. C. Ridding , G. Dirnberger , and J. C. Rothwell . 1998. “The Effects of Transcranial Magnetic Stimulation Over the Dorsolateral Prefrontal Cortex on Suppression of Habitual Counting During Random Number Generation.” Brain 121, no. 8: 1533–1544. 10.1093/brain/121.8.1533. 9712014

[dys70030-bib-0039] Jahanshahi, M. , T. Saleem , A. K. Ho , G. Dirnberger , and R. Fuller . 2006. “Random Number Generation as an Index of Controlled Processing.” Neuropsychology 20, no. 4: 391–399. 10.1037/0894-4105.20.4.391. 16846257

[dys70030-bib-0040] Kaga, Y. , R. Ueda , M. Tanaka , et al. 2020. “Executive Dysfunction in Medication‐Naïve Children With ADHD: A Multi‐Modal fNIRS and EEG Study.” Brain and Development 42, no. 8: 555–563. 10.1016/j.braindev.2020.05.007. 32532641

[dys70030-bib-0093] Kendall, M. G. 1976. Time‐Series. 2nd ed. Griffin.

[dys70030-bib-0041] Lambek, R. , R. Tannock , S. Dalsgaard , A. Trillingsgaard , D. Damm , and P. H. Thomsen . 2011. “Executive Dysfunction in School‐Age Children With ADHD.” Journal of Attention Disorders 15, no. 8: 646–655. 10.1177/1087054710370935. 20858784

[dys70030-bib-0042] Lezak, M. D. 1982. “The Problem of Assessing Executive Functions.” International Journal of Psychology 17: 281–297. 10.1080/00207598208247445.

[dys70030-bib-0043] Lonergan, A. , C. Doyle , C. Cassidy , et al. 2019. “A Meta‐Analysis of Executive Functioning in Dyslexia With Consideration of the Impact of Comorbid ADHD.” Journal of Cognitive Psychology 31, no. 7: 725–749. 10.1080/20445911.2019.1669609.

[dys70030-bib-0044] López‐Zamora, M. , N. Porcar‐Gozalbo , I. López‐Chicheri García , and A. Cano‐Villagrasa . 2025. “Linguistic and Cognitive Abilities in Children With Dyslexia: A Comparative Analysis.” European Journal of Investigation in Health, Psychology and Education 15, no. 3: 37. 10.3390/ejihpe15030037. 40136777 PMC11941291

[dys70030-bib-0045] Lunt, L. , J. Bramham , R. G. Morris , et al. 2012. “Prefrontal Cortex Dysfunction and “Jumping to Conclusions”: Bias or Deficit?” Journal of Neuropsychology 6, no. 1: 65–78. 10.1111/j.1748-6653.2011.02005.x. 22257612

[dys70030-bib-0046] McLoughlin, D. , G. Fitzgibbon , and V. Young . 1994. Adult Dyslexia: Assessment, Counselling and Training. Whurr.

[dys70030-bib-0047] Meltzer, L. 1991. “Problem‐Solving Strategies and Academic Performance in Learning Disabled Students: Do Subtypes Exist?” In Subtypes of Learning Disabilities: Theoretical Perspectives and Research, edited by L. V. Feagans , E. J. Short , and L. J. Meltzer , 163–188. Lawrence Erlbaum Associates.

[dys70030-bib-0048] Menghini, D. , A. Finzi , G. A. Carlesimo , and S. Vicari . 2011. “Working Memory Impairment in Children With Developmental Dyslexia: Is It Just a Phonological Deficity?” Developmental Neuropsychology 36, no. 2: 199–213. 10.1080/87565641.2010.549868. 21347921

[dys70030-bib-0049] Miyake, A. , N. P. Friedman , M. J. Emerson , A. H. Witzki , A. Howerter , and T. D. Wager . 2000. “The Unity and Diversity of Executive Functions and Their Contributions to Complex “Frontal Lobe” Tasks: A Latent Variable Analysis.” Cognitive Psychology 41: 49–100. 10.1006/cogp.1999.0734. 10945922

[dys70030-bib-0050] Monsell, S. 1996. “Control of Mental Processes.” In Unsolved Mysteries of the Mind: Tutorial Essays in Cognition, edited by V. Bruce , 93–148. Erlbaum.

[dys70030-bib-0051] Monsell, S. 2003. “Task Switching.” Trends in Cognitive Sciences 7, no. 3: 134–140. 10.1016/s1364-6613(03)00028-7. 12639695

[dys70030-bib-0052] Morris, N. , and D. M. Jones . 1990. “Memory Updating in Working Memory: The Role of the Central Executive.” British Journal of Psychology 81, no. 2: 111–121. 10.1111/j.2044-8295.1990.tb02349.x.

[dys70030-bib-0053] Nicolson, R. I. , and A. J. Fawcett . 1990. “Automaticity: A New Framework for Dyslexia Research.” Cognition 35: 159–182. 10.1016/0010-0277(90)90013-a. 2354611

[dys70030-bib-0054] Nicolson, R. I. , A. J. Fawcett , and P. Dean . 1995. “Time Estimation Deficits in Developmental Dyslexia: Evidence of Cerebellar Involvement.” Proceedings of the Royal Society of London, Series B: Biological Sciences 259, no. 1354: 43–47. 10.1098/rspb.1995.0007. 7700875

[dys70030-bib-0055] Nigg, J. T. 2000. “On Inhibition/Disinhibition in Developmental Psychopathology: Views From Cognitive and Personality Psychology and a Working Inhibition Taxonomy.” Psychological Bulletin 126, no. 2: 220–246. 10.1037/0033-2909.126.2.220. 10748641

[dys70030-bib-0056] Norman, D. A. , and T. Shallice . 1986. “Attention to Action: Willed and Automatic Control of Behaviour.” In Consciousness and Self‐Regulation: Advances in Research and Theory, edited by R. J. Davidson , G. E. Schwartz , and D. Shapiro , vol. 4, 1–18. Plenum Press.

[dys70030-bib-0057] Olusanya, B. O. , T. Smythe , F. A. Ogbo , M. K. C. Nair , M. Scher , and A. C. Davis . 2023. “Global Prevalence of Developmental Disabilities in Children and Adolescents: A Systematic Umbrella Review.” Frontiers in Public Health 11: 1122009. 10.3389/fpubh.2023.1122009. 36891340 PMC9987263

[dys70030-bib-0058] Oomens, W. , J. H. Maes , F. Hasselman , and J. I. Egger . 2015. “A Time Series Approach to Random Number Generation: Using Recurrence Quantification Analysis to Capture Executive Behavior.” Frontiers in Human Neuroscience 9: 319. 10.3389/fnhum.2015.00319. 26097449 PMC4456862

[dys70030-bib-0059] Peters, M. , T. Giesbrecht , M. Jelicic , and H. Merckelbach . 2007. “The Random Number Generation Task: Psychometric Properties and Normative Data of an Executive Function Task in a Mixed Sample.” Journal of the International Neuropsychological Society 13, no. 4: 626–634. 10.1017/S1355617707070786. 17521494

[dys70030-bib-0060] Phillips, L. H. 1997. “Do “Frontal Tests” Measure Executive Function? Issues of Assessment and Evidence From Fluency Tests.” In Methodology of Frontal and Executive Function, edited by P. M. A. Rabbitt , 191–213. Psychology Press.

[dys70030-bib-0061] Phillips, L. H. , R. Bull , E. Adams , and L. Fraser . 2002. “Positive Mood and Executive Function: Evidence From Stroop and Fluency Tasks.” Emotion 2, no. 1: 12–22. 10.1037/1528-3542.2.1.12. 12899364

[dys70030-bib-0062] Proios, H. , S. S. Asaridou , and P. Brugger . 2008. “Random Number Generation in Patients With Aphasia: A Test of Executive Functions.” Acta Neuropsychologica 6, no. 2: 157–168. 10.5167/uzh-9127.

[dys70030-bib-0063] Rabbitt, P. 1997. “Introduction: Methodologies and Models in the Study of Executive Function.” In Methodology of Frontal and Executive Function, 1–38. Psychology Press.

[dys70030-bib-0064] Rabinowitz, M. 1970. “Characteristic Sequential Dependencies in Multiple‐Choice Situations.” Psychological Bulletin 74: 141–148. 10.1037/h0029551.

[dys70030-bib-0065] Reiter, A. , O. Tucha , and K. W. Lange . 2005. “Executive Functions in Children With Dyslexia.” Dyslexia 10: 116–131. 10.1002/dys.289. 15918370

[dys70030-bib-0066] Rose, J. 2009. Identifying and Teaching Children With Dyslexia and Other Literacy Difficulties. https://www.thedyslexia‐spldtrust.org.uk/media/downloads/inline/the‐rose‐report.1294933674.pdf.

[dys70030-bib-0067] Shannon, C. E. 1948. “A Mathematical Theory of Communication.” Bell System Technical Journal 27: 379–423.

[dys70030-bib-0068] Siegel, L. S. 2006. “Perspectives on Dyslexia.” Paediatrics & Child Health 11, no. 9: 581–587. 10.1093/pch/11.9.581. 19030329 PMC2528651

[dys70030-bib-0069] Smith‐Spark, J. H. , and J. E. Fisk . 2007. “Working Memory Functioning in Developmental Dyslexia.” Memory 15, no. 1: 34–56. 10.1080/09658210601043384. 17479923

[dys70030-bib-0070] Smith‐Spark, J. H. , and R. Gordon . 2022. “Automaticity and Executive Abilities in Developmental Dyslexia: A Theoretical Review.” Brain Sciences 12: 446. 10.3390/brainsci12040446. 35447978 PMC9030885

[dys70030-bib-0071] Smith‐Spark, J. H. , L. A. Henry , D. J. Messer , E. Edvardsdottir , and A. P. Zięcik . 2016. “Executive Functions in Adults With Developmental Dyslexia.” Research in Developmental Disabilities 53‐54: 323–341. 10.1016/j.ridd.2016.03.001. 26970859

[dys70030-bib-0072] Smith‐Spark, J. H. , L. A. Henry , D. J. Messer , and A. P. Zięcik . 2017. “Verbal and Non‐Verbal Fluency in Adults With Developmental Dyslexia: Phonological Processing or Executive Control Problems?” Dyslexia 23, no. 3: 234–250. 10.1002/dys.1558. 28493359

[dys70030-bib-0074] Snowling, M. J. , C. Hulme , and K. Nation . 2020. “Defining and Understanding Dyslexia: Past, Present and Future.” Oxford Review of Education 46, no. 4: 501–513. 10.1080/03054985.2020.1765756. 32939103 PMC7455053

[dys70030-bib-0073] Snowling, M. , and C. Hulme . 2024. “Do We Really Need a New Definition of Dyslexia? A Commentary.” Annals of Dyslexia 74, no. 3: 355–362. 10.1007/s11881-024-00305-y. 38526759 PMC11413107

[dys70030-bib-0075] Spatt, J. , and G. Goldenberg . 1993. “Components of Random Generation by Normal Subjects and Patients With Dysexecutive Syndrome.” Brain and Cognition 23, no. 2: 231–242. 10.1006/brcg.1993.1057. 8292327

[dys70030-bib-0076] Thompson, P. A. , C. Hulme , H. M. Nash , D. Gooch , E. Hayiou‐Thomas , and M. J. Snowling . 2015. “Developmental Dyslexia: Predicting Individual Risk.” Journal of Child Psychology and Psychiatry, and Allied Disciplines 56, no. 9: 976–987. 10.1111/jcpp.12412. 25832320 PMC4672694

[dys70030-bib-0077] Torgeson, J. K. , and T. Goldman . 1977. “Verbal Rehearsal and Short‐Term Memory in Reading‐Disabled Children.” Child Development 48: 56–60. 844361

[dys70030-bib-0078] Towse, J. N. 1998. “On Random Generation and the Central Executive of Working Memory.” British Journal of Psychology 89, no. 1: 77–101. 10.1111/j.2044-8295.1998.tb02674.x. 9532724

[dys70030-bib-0079] Towse, J. N. , and D. Neil . 1998. “Analyzing Human Random Generation Behavior: A Review of Methods Used and a Computer Program for Describing Performance.” Behavior Research Methods, Instruments, & Computers 30: 583–591. 10.3758/BF03209475.

[dys70030-bib-0080] Towse, J. N. , and J. D. Valentine . 1997. “Random Generation of Numbers: A Search for Underlying Processes.” European Journal of Cognitive Psychology 9, no. 4: 381–400. 10.1080/713752566.

[dys70030-bib-0081] Troyer, A. K. , M. Moscovitch , and G. Winocur . 1997. “Clustering and Switching as Two Components of Verbal Fluency: Evidence From Younger and Older Healthy Adults.” Neuropsychology 11, no. 1: 138–146. 10.1037/0894-4105.11.1.138. 9055277

[dys70030-bib-0082] Turner, M. 1997. Psychological Assessment of Dyslexia. Whurr.

[dys70030-bib-0083] Varvara, P. , C. Varuzza , A. C. Sorrentino , S. Vicari , and D. Menghini . 2014. “Executive Functions in Developmental Dyslexia.” Frontiers in Human Neuroscience 8: 120. 10.3389/fnhum.2014.00120. 24639640 PMC3945518

[dys70030-bib-0084] Vellutino, F. R. 1979. Dyslexia: Theory and Research. MIT Press.

[dys70030-bib-0085] Vellutino, F. R. , J. M. Fletcher , M. J. Snowling , and D. M. Scanlon . 2004. “Specific Reading Disability (Dyslexia): What Have We Learned in the Past Four Decades?” Journal of Child Psychology and Psychiatry 45, no. 1: 2–40. 10.1046/j.0021-9630.2003.00305.x. 14959801

[dys70030-bib-0086] Wang, J. , S. Huo , K. C. Wu , J. Mo , W. L. Wong , and U. Maurer . 2022. “Behavioral and Neurophysiological Aspects of Working Memory Impairment in Children With Dyslexia.” Scientific Reports 12, no. 1: 12571. 10.1038/s41598-022-16729-8. 35869126 PMC9307804

[dys70030-bib-0087] Wechsler, D. 1993. The Wechsler Objective Reading Dimensions. Psychological Corporation.

[dys70030-bib-0088] Wechsler, D. 2010. Wechsler Adult Intelligence Scale. 4th ed. Psychological Corporation.

[dys70030-bib-0089] Welsh, M. C. , B. F. Pennington , and D. B. Groisserc . 1991. “A Normative‐Developmental Study of Executive Function: A Window on Prefrontal Function in Children.” Developmental Neuropsychology 7: 131–149. 10.1080/87565649109540483.

[dys70030-bib-0090] Wilcockson, T. D. W. , D. Mardanbegi , P. Sawyer , H. Gellersen , B. Xia , and T. J. Crawford . 2019. “Oculomotor and Inhibitory Control in Dyslexia.” Frontiers in Systems Neuroscience 12, no. 6: 66. 10.3389/fnsys.2018.00066. 30687026 PMC6338055

[dys70030-bib-0091] Williams, M. A. , S. A. Moss , J. L. Bradshaw , and N. J. Rinehart . 2002. “Random Number Generation in Autism.” Journal of Autism and Developmental Disorders 32: 43–47. 10.1023/a:1017904207328. 11916332

